# Supplementary material for: Contribution of three tRNA modification enzymes to Proteus mirabilis fitness and catheter-associated urinary tract infection
Source: Infect Immun. 2026 Apr 27;94(6):e00722-25. doi: 10.1128/iai.00722-25 (PMC13248701; doi:10.1128/iai.00722-25)
Supplement: Supplemental Figures — Figures S1 to S4. [file iai.00722-25-s0001.pdf]

Supplemental Figure 1. The loss of *dusB* causes a competitive growth defect during co-culture in multiple media types. (A–B) Fitness defects of the *dusB::Kan* mutant were assessed by inoculating LB or AUM, respectively, with a 1:1 mixture of wild-type and the mutant, and CFUs were assessed by differential plating at hourly time points over 7 hours. Competitive indices (CI) for *dusB::Kan* were then calculated, revealing a CI < 1 consistent with decreased fitness during co-challenge. The dotted line marks a CI of 1 (equal fitness). **(C)** The impact of plasmid-based complementation of the *dusB::Kan* mutant was assessed, with *dusB pGEN-dusB* reverting to wild-type fitness levels by both CFUs and CI. Data are shown as mean ± SD with at least 3 biological replicates performed for each; significance was assessed via two-way ANOVA with multiple comparison correction or Wilcoxon signed-rank test using GraphPad Prism v10.5.

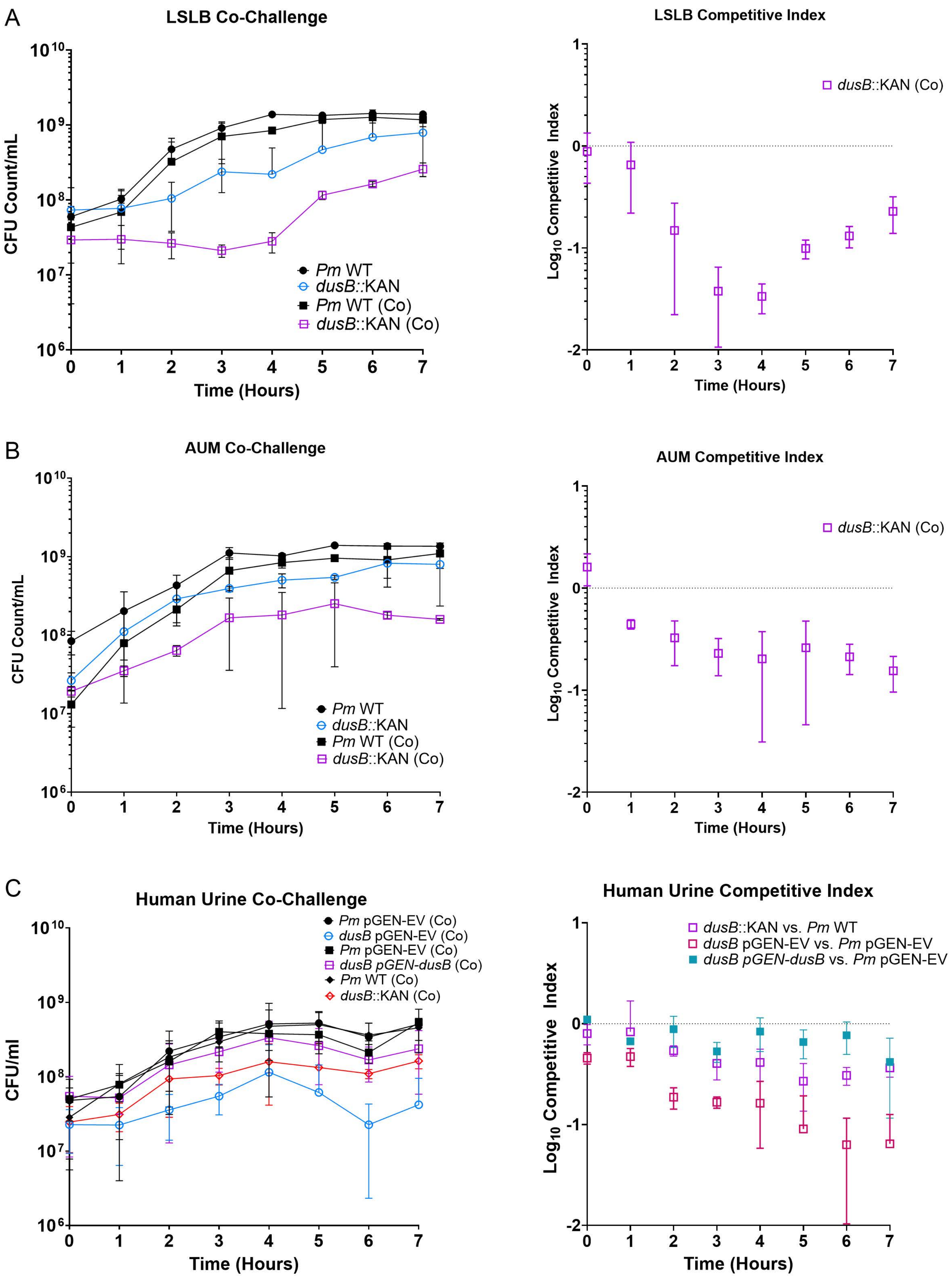

A
LSLB Co-Challenge
CFU Count/mL
Time (Hours)
Pm WT
dusB::KAN
Pm WT (Co)
dusB::KAN (Co)
LSLB Competitive Index
dusB::KAN (Co)
Log10 Competitive Index
Time (Hours)
B
AUM Co-Challenge
CFU Count/mL
Time (Hours)
Pm WT
dusB::KAN
Pm WT (Co)
dusB::KAN (Co)
AUM Competitive Index
dusB::KAN (Co)
Log10 Competitive Index
Time (Hours)
C
Human Urine Co-Challenge
CFU/ml
Time (Hours)
Pm pGEN-EV (Co)
dusB pGEN-EV (Co)
Pm pGEN-EV (Co)
dusB pGEN-dusB (Co)
Pm WT (Co)
dusB::KAN (Co)
Human Urine Competitive Index
dusB::KAN vs. Pm WT
dusB pGEN-EV vs. Pm pGEN-EV
dusB pGEN-dusB vs. Pm pGEN-EV
Log10 Competitive Index
Time (Hours)

Supplemental Figure 2. Swimming motility of *P. mirabilis* wild-type and *dus* mutants on soft agar. Representative images of swimming halos on 0.25% agar plates after 18 hours at 30°C. (A) *P. mirabilis* HI4320 wild-type, (B) *dusA::Kan*, (C) *dusB::Kan*, (D) *dusC::Kan*, (E) *Pm* pGEN-EV, (F) *dusB* pGEN-EV, and (G) *dusB* pGEN-*dusB*.

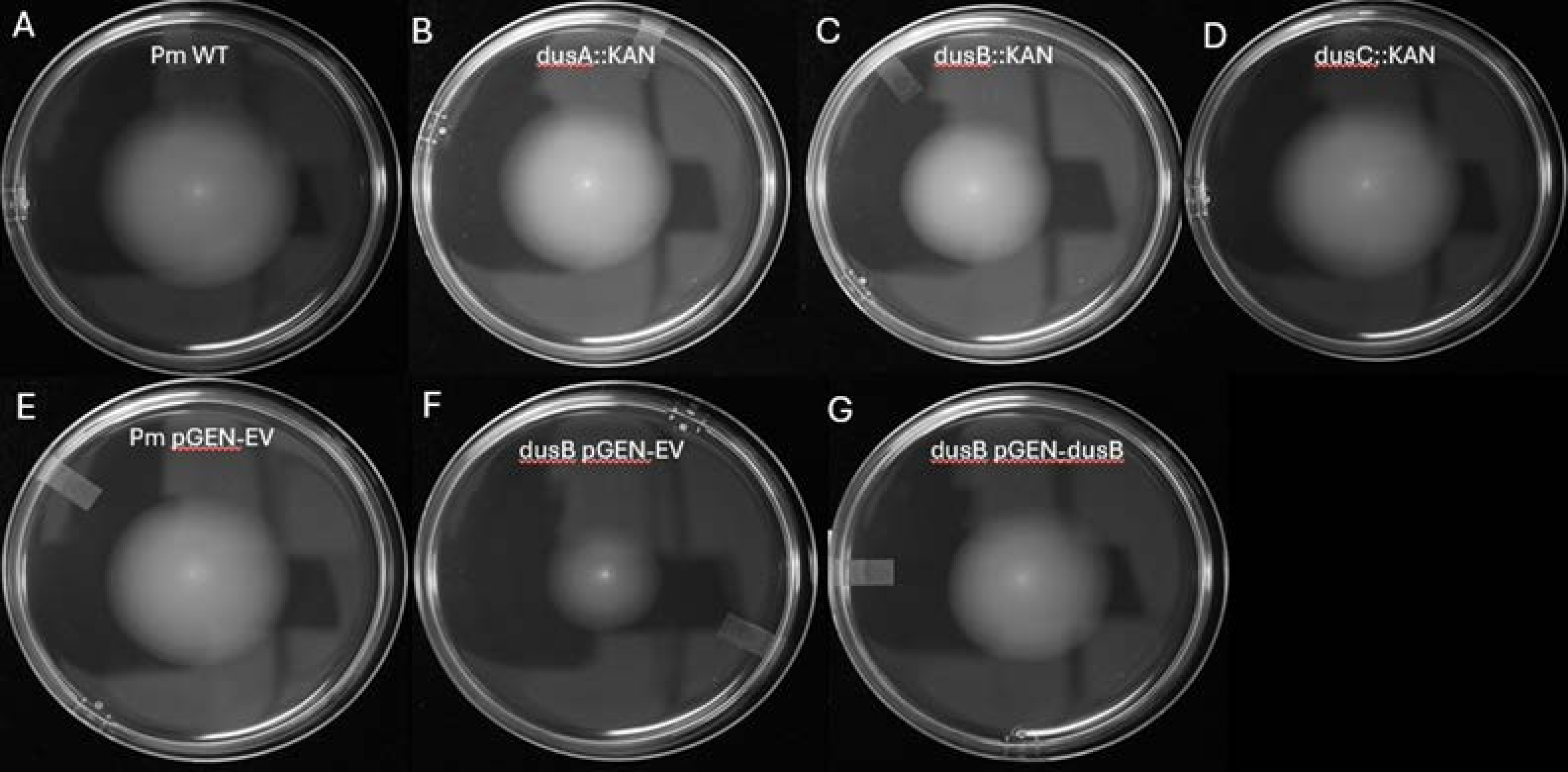
A
Pm WT
B
dusA::KAN
C
dusB::KAN
D
dusC::KAN
E
Pm pGEN-EV
F
dusB pGEN-EV
G
dusB pGEN-dusB

Supplemental Figure 3. Swarming morphology of *P. mirabilis* wild-type and *dus* mutants on swarm agar. Representative images of *P. mirabilis* HI4320 wild-type (A), *dusA::Kan* (B), *dusB::Kan* (C), and *dusC::Kan* (D) grown on 1.5% swarm agar after 18 hours at 37°C.

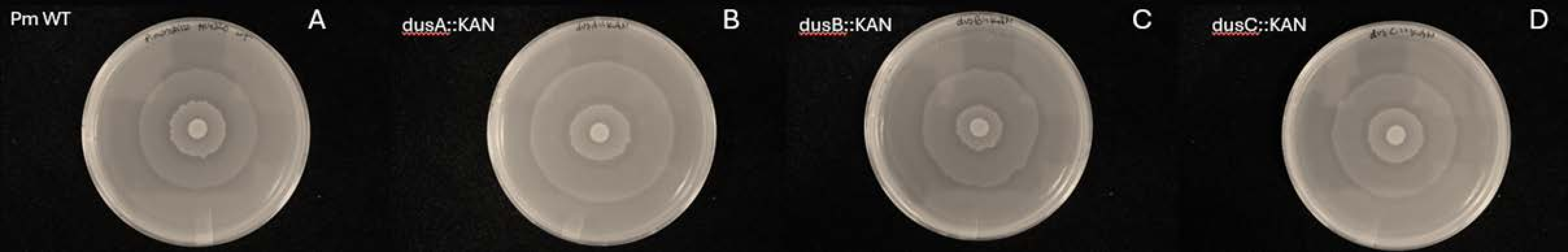
Pm WT
A
dusA::KAN
B
dusB::KAN
C
dusC::KAN
D

Supplemental Figure 4. DusB contributes to *P. mirabilis* fitness as early as 48h post-infection. CBA/J mice were transurethrally catheterized and co-infected with 1x10^5 CFUs of a 1:1 mix of WT *P. mirabilis* and *dusB::Kan*. Bacterial burdens were enumerated from urine, bladder, kidneys, and spleen after 48 h. Left panel shows CFU/g or CFU/ml for WT and *dusB::Kan* from the same mouse connected by a black line. Right panel shows calculated competitive indices (mutant/WT). Statistical significance was determined by Wilcoxon signed-rank test.

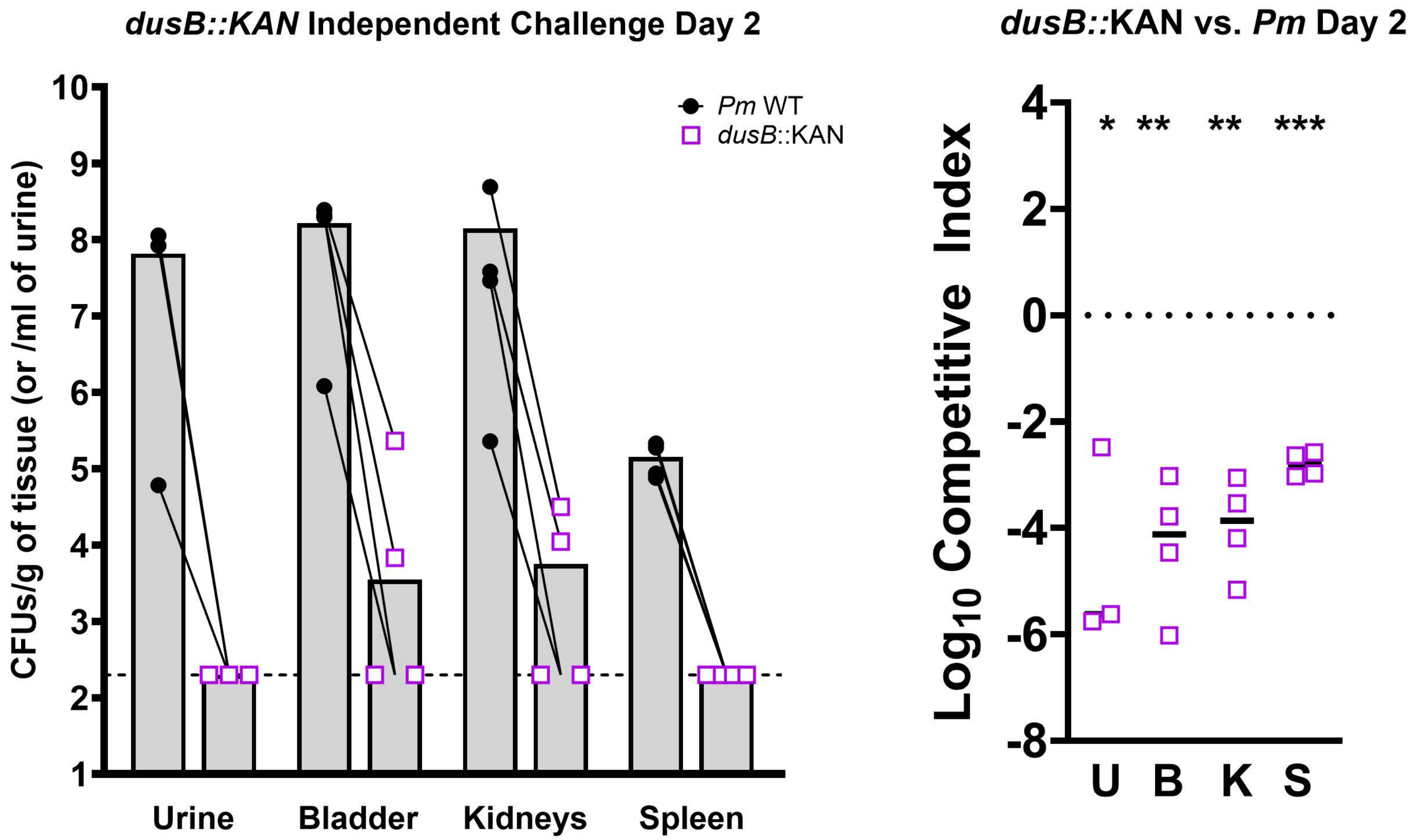
dusB::KAN Independent Challenge Day 2
Pm WT
dusB::KAN
CFUs/g of tissue (or /ml of urine)
10
9
8
7
6
5
4
3
2
1
Urine
Bladder
Kidneys
Spleen
dusB::KAN vs. Pm Day 2
Log10 Competitive Index
4
2
0
-2
-4
-6
-8
*
**
**
***
U
B
K
S
